# Mitochondrial genome assembly and comparative analysis of decaploid *Camellia hainanica*

**DOI:** 10.3389/fpls.2025.1556379

**Published:** 2025-06-30

**Authors:** Shihui Zhang, Yuyan Zhang, Sheng Luo, Jie Gao, Haiyan Hu, Jinping Liu, Wenqiang Wu, Jian Wang, Xiaolong Huang, Hanggui Lai, Dongyi Huang

**Affiliations:** ^1^ School of Tropical Agriculture and Forestry, Hainan University, Danzhou, China; ^2^ School of Life and Health Sciences, Hainan University, Haikou, China; ^3^ School of Breeding and Multiplication (Sanya Institute of Breeding and Multiplication), Hainan University, Sanya, China

**Keywords:** decaploid *Camellia hainanica*, mitochondrial genome, comparative analysis, RNA editing, analysis system development

## Abstract

**Introduction:**

Decaploid *Camellia hainanica* is a new tea oil *Camellia* species discovered in recent years that is unique to Hainan. This species has high nutritional and medicinal value and shows strong adaptability in the growth process. Mitochondria play an important role in plant cells and have an independent genetic system. Therefore, assembling and annotating the mitochondrial genome function of decaploid *C. hainanica* is of great significance.

**Methods:**

This study successfully assembled the mitochondrial genome of decaploid *C. hainanica* and comprehensively annotated its functional genes using the Nanopore sequencing platform.

**Results:**

Results showed that the mitochondrial genome is 902,617 bp in length, with a typical circular structure and a guanine–cytosine content of 45.79%. The genome encodes 64 protein-coding genes and contains a total of 76 genes, including 40 mRNA, 32 tRNA, 3 rRNA, and 1 pseudogene. Tetranucleotide repeats accounted for 38.60% of the simple sequence repeats. Only two genes, *atp6* and *sdh4*, had a Ka/Ks ratio <1, whereas the Pi value of the *sdh3* gene had a maximum of 0.00374 in these regions, suggesting that the *sdh3* gene can be used as a molecular marker for the analysis of the mitochondrial genome of *C. hainanica*. From the relative synonymous codon usage (RSCU) analysis, 29 codons had RSCU values >1, 27 of which (93%) ended in A or U, indicating a bias for A/U endings is present in *C. hainanica*. During RNA editing, 48.24% (260 loci) of amino acids were changed from hydrophilic to hydryophobic, resulting in an increase in the hydrophobicity of the protein. Comparative analysis identified 34 homologous fragments between the mitochondrial and chloroplast genomes, with the longest fragment being 9,572 bp in length. Phylogenetic analysis of the genomes showed that the Hainanese and Vietnamese varieties of tea oil *Camellia* are sister species.

**Discussion:**

Results confirmed that the mitochondrial genomes of Hainanese and Vietnamese tea oil *Camellia* underwent gene rearrangement. Results also provided key data support for the utilization and conservation of tea oil germplasm resources and the breeding of varieties and are of great significance for promoting genetic evolution research, genetic breeding, and identification of tea oil *Camellia*.

## 1 Introduction

Tea oil Camellia (*Camellia oleifera Abel.*) is a small evergreen tree or shrub with a high seed oil content and economic cultivation value. Tea oil *Camellia* originates in China and has a long history of cultivation in Hainan, where it is one of the island’s traditional plants (Liang et al., 2024).

Recent advances in *Camellia oleifera* genomics have revealed significant progress in understanding its genetic architecture and agronomic traits. While previous studies predominantly focused on diploid assemblies (Ye et al., 2023), emerging research highlights the imperative to investigate polyploid genomes. Current findings demonstrate that wild *C. oleifera* exists as a heterozygous hexaploid species characterized by elevated genomic heterozygosity (0.82%) and substantial repetitive element content (∼68% of genome), as evidenced by Haoxing Xie (Xie et al., 2024) through PacBio HiFi sequencing. This breakthrough enabled the identification of 21,437 SSR markers and the characterization of cold-responsive genes including CBF and ICE1 transcription factors.

Notably, our investigation of Hainan Island germplasm uncovered a unique ploidy distribution pattern dominated by decaploid (2n=10x=150) and octaploid (2n=8x=120) cytotypes. Phylogenetic analyses suggest these polyploid complexes likely originated through adaptive radiation under the tropical monsoon climate regime, with the decaploid form representing an endemic lineage showing distinct evolutionary divergence (FST > 0.25). The genomic complexity of these cytotypes, particularly the decaploid’s 15 Gb genome featuring 12.3% tandem repeats, presents both challenges and opportunities for resolving paleopolyploidization events through third-generation sequencing approaches.

These findings substantially expand the genomic resources for *Camellia* species while providing molecular tools for marker-assisted selection. The Hainan decaploid accessions, with their exceptional environmental adaptation mechanisms, hold significant promise for elucidating polyploid genome evolution and developing stress-resilient cultivars. Mitochondria harbor their own genetic code and protein translation system. Their DNA, known as mtDNA, encodes key components of cellular energy supply and participates in essential biological processes. Unlike the plant chloroplast genome, the mitochondrial genome exhibits remarkable diversity due to lineage-specific evolutionary development (Wideman et al., 2020; Butenko et al., 2024). This diversity enables mitochondria to play crucial roles in energy conversion, fatty acid synthesis, amino acid metabolism, and stress responses, thereby enhancing their adaptability to changing environmental conditions and contributing to the adaptive evolution of plants. Although mtDNA is typically described as a circular molecule, diverse structures have been identified, including linear conformations, branching structures, and numerous smaller circular molecules (Sloan, 2013; Gualberto et al., 2014). These complex and varied structures harbor a vast amount of genetic information, which is invaluable for resolving species classification, accurate identification, and elucidating the evolutionary trajectories of species.

Most previous studies on *C. hainanica* focus on leaf characteristics and pollen spore analysis. However, the morphological characteristics of the plant are greatly affected by environmental changes. With advances in molecular biotechnology, many researchers have employed molecular biomarker technology and DNA sequencing methods to study species classification. The first complete assembly of the mitochondrial genome of the diploid tea oil species *C. gigantocarpa* was completed in 2022 (Lu et al., 2022). Using the PacBioHi-Fi and Hi-C sequencing technologies, the mitochondrial genome of *C. gigantocarpa* was successfully assembled. The proportion of repetitive sequences in the *C. gigantocarpa* mitochondrial genome is as high as 20.81%, comparable to that of *C. sinensis* (22.15%), but much higher than that of *Arabidopsis thaliana* (4.96%). This significantly increases the size of the mitochondrial genome of tea oil *Camellia*. In their analysis of the hexaploid tea oil variety *C. oleifera* cv. *Huashuo*, researchers have successfully assembled the full mitochondrial genome of this tea oil *Camellia* variety for the first time using second-generation sequencing technology. The study revealed a tea oil *Camellia* mitochondrial genome with a circular structure consisting of 709,596 bp and successfully annotated 74 genes in this genome (Gu et al., 2024). In a study of the mitochondrial genome of congener *C. assamica*, Rawal et al. (2020) obtained the complete *C. assamica* mitochondrial genome by de-redundancy assembly of *587,142* filtered mitochondrial read sequences, obtaining a total length of 707,441 bp. The overall guanine–cytosine (GC) content was 45.75%, with a total of 66 annotated genes, including 35 protein-coding genes (PCGs), 29 tRNA, and 2 rRNA (Rawal et al., 2020). Li et al. (2024) studied the complete assembly and annotation of the mitochondrial genomes of 4 species within the tea oil *Camellia* lineage, demonstrating that the mitochondrial genome consists of closed-loop DNA molecules ranging in size from 850,836 bp (*C. nitidissma*) to 109,8121 bp (*C. tianeensis*) (Li et al., 2024). In a study on *C. duntsa*, Li et al. (2023b) demonstrated that its mitochondrial genome consists of 1,081,996 bp and 81 genes, including 1 pseudogene, 3 rRNA genes, 30 tRNA genes, and 47 PCGs (Li et al., 2023b). Although studies on the mitochondrial genomes of hexaploid tea oil *Camellia* and related species have been reported, reports regarding the mitochondrial genomes of higher ploidy tea oil *Camellia* varieties are currently lacking.

Based on this in-depth study of its mitochondrial genome, we aim to provide a scientific basis for the breeding and genetic improvement of tea oil *Camellia*. At the same time, comparative genomic analysis of decaploid *C. hainanica* with similar species can not only reveal the genetic differences between decaploid *C. hainanica* and other tea oil *Camellia* varieties but can also provide important data-based support for the utilization and protection of tea oil *Camellia* genetic resources, which can help promote the study of genetic evolution of the species and scientific identification in tea oil *Camellia* breeding.

## 2 Materials and methods

The *C. hainanica* in this study was sampled from Huishan Township, Qionghai City, Hainan Province (longitude 110°18′20″E, latitude 19°5′18″N, elevation 82.00 m), with the crops having been cultivated under natural conditions. The young leaves of perennial decaploid *C. hainanica* were collected, placed into liquid nitrogen for snap freezing, and stored in a −80°C freezer before being sent to Benagen for sequencing. The Plant Genomic DNA kit DP305 (Tiangen Biotech, Beijing, China) was used in this study. DNA purity was measured using a 1.0% agarose gel. To obtain an accurate full-length mitochondrial genome, short- and long-read sequencing technologies were combined in this study. The short-read sequencing platform used was Illumina NovaSeq 6000 (Illumina, San Diego, CA, USA), with a paired-end sequencing read length of 150 bp. The fastp (version 0.20.0; https://github.com/OpenGene/fastp) software was used to filter raw data and obtain high-quality reads. The long-read sequencing platform used was Nanopore PromethION (Nanopore, Oxford, UK), and the sequencing data were filtered by the filtlong software (version 0.2.1; https://link.zhihu.com/?target=https%3A//github.com/rrwick/Filtlong).

### 2.1 Mitochondrial genome assembly and annotation

Plant mitochondrial genes (coding sequence [CDS], rRNA) are highly conserved. By employing the third-generation alignment software minimap2 (Li, 2018), this characteristic was used to compare the third-generation data to the reference gene sequences (plant mitochondrial core genes, https://github.com/xul962464/plant_mt_ref_gene) and screened for sequences with an alignment length of >50 bp as candidate sequences for comparison. From these sequences, those with a larger number of aligned genes (sequences containing multiple core genes) and a higher alignment quality (the core genes covered were more complete) were selected as seed sequences. Next, minimap2 was used to align the original third-generation sequencing data to the seed sequences and screen for sequences with an overlap of >1 kb, which were then added to the seed sequences. Iterative alignment of the original data to the seed sequences was conducted, thus obtaining the complete third-generation sequencing data of the mitochondrial genome. Then, the third-generation assembly software canu (Koren et al., 2017) was used to correct the resulting third-generation data, and the corrected third-generation data were spliced using the default parameters of SPAdes (version 3.15.4, https://github.com/ablab/spades#metapv). The splicing results were visualized and manually adjusted using the Bandage (version 0.8.1) (Wick et al., 2015; https://github.com/rrwick/Bandage) software. Due to the complex physical structure of the mitochondrial genome that consists of multiple subloops, or even nonloops, the corrected third-generation sequencing data were aligned to the contig obtained from SPAdes (Andrey et al., 2020) using minimap2 to manually determine the branching direction, thereby obtaining the final assembly results.

Mitochondrial annotation was performed using the following steps:

1. Encoded proteins and rRNA were aligned to published and ref plant mitochondrial sequences using BLAST, with further manual adjustments made for closely related species.
2. tRNA was annotated using tRNAscanSE (5) (http://lowelab.ucsc.edu/tRNAscan-SE/).
3. Open reading frames were annotated using the Open Reading Frame Finder (http://www.ncbi.nlm.nih.gov/gorf/gorf.html) by setting the minimum length to 102 bp to exclude redundant sequences and sequences that overlap with known genes. Sequences >300 bp in length were annotated against the nr library.
4. RNA editing sites were originally predicted using PmtREP (http://112.86.217.82:9919/#/tool/alltool/detail/336). The final annotation results were obtained after checking and manually correcting the obtained results.

### 2.2 Synonymous codon usage bias analysis

The mitochondrial genome codon composition of C. hainanica was screened for unique CDS and calculated using a script written in Perl(http://cloud.genepioneer.com:9929/#/tool/alltool/detail/214). Its calculation method is: (the number of a certain codon encoding an amino acid/the number of all codons encoding that amino acid)/(1/the number of codon types encoding that amino acid), that is, (the actual usage frequency of the codon/the theoretical usage frequency of the codon).

### 2.3 Identification of RNA editing sites

The RNA sequencing data were aligned to the CDS (Coding DNA Sequence) sequences by utilizing Bowtie2 (version 2.3.5.1; https://github.com/BenLangmead/bowtie2)(Langmead and Salzberg, 2012), and subsequently processed using samtools(https://github.com/samtools) for further analysis. The software bcftools (1.9-170) (https://github.com/samtools/bcftools)was then used to identify sites where single-nucleotide polymorphisms existed between the sequencing data and the genome, which served as potential RNA editing sites.

### 2.4 Repeated sequence analysis

Repeated sequences include simple sequence repeats (SSRs), tandem repeats, and dispersed repeats. SSRs were identified using the misa software (version 1.0, parameters: 1-10 2-5 3-4 4-3 5-3 6-3, https://webblast.ipk-gatersleben.de/misa/), tandem repeats were identified using the trf software (trf409, parameters: 2 7 7 80 10 50 2000 -f -d -m, http://tandem.bu.edu/trf/trf. submit.options.html), and dispersed repeats were identified using BLASTn software (version 2.10.1, parameters: -word_size 7, E-value 1e-5, de-redundancy, tandem duplicates were removed) and visualized using circos v0.69-5.

### 2.5 Ka/Ks and Pi analyses

Binary grouping of the higher-order analyzed species was conducted to perform Ka/Ks analysis. Homologous gene pairs were then extracted, and the homologous gene pairs were aligned using the mafft version 7.427 (https://mafft.cbrc.jp/alignment/software/) software. After alignment, the KaKs_Calculator version 2.0 (Zhang, 2022) software was used to calculate the Ka and Ks values of each gene pair (https://sourceforge.net/projects/kakscalculator2/), with the MLWL calculation method selected.

Global alignment of homologous gene sequences from different species was performed using the mafft software (version 7.427, –automode), and the Pi values for each gene were calculated using dnasp5.

### 2.6 Phylogenetic and collinearity analyses

Phylogenetic analysis of the mitochondria in this *Camellia* genus was conducted as part of this study. Twenty-five plant mitochondrial genome sequences (17 from the family Theaceae) were downloaded from the National Center for Biotechnology Information database, with the genera *Brassica*, *Aquilaria*, *Dalbergia*, *Hevea*, *Olea*, and *Cocos* as outgroups. Extract the CDS (Coding DNA Sequences) that are shared by 70% or more of the species for the construction of the phylogenetic tree. and multisequence alignment of interspecies sequences was carried out using the mafft software (v7.427, –auto mode). The aligned sequences were joined head to tail and trimmed with trimAl (version 1.4.rev15) (parameter: -gt 0.7) (Capella-Gutiérrez et al., 2009; https://github.com/inab/trimal). After trimming, the software jmodeltest-2.1.10 was used for model prediction. The model was determined to be of the general time reversible type. The maximum likelihood evolutionary tree was constructed using the RAxML version 8.2.10 (https://cme.h-its.org/exelixis/software.html) software with the GTRGAMMA model selected and bootstrap=1000.

Collinearity analysis was performed using the nucmer (4.0.0beta2) (https://github.com/mummer4/mummer) software, with the –maxmatch parameter used for genomic comparison between other sequences and assembled sequences so as to generate dot-plots.

### 2.7 Analysis of mitochondrial and chloroplast homologous fragments

Homologous sequences between chloroplasts and mitochondria were found using the BLAST software, with the E-value set to 1e-5 and similarity set to not fall <70%.

## 3 Results

### 3.1 Decaploid *C. hainanica* mitochondrial genome

**Figure 1:**
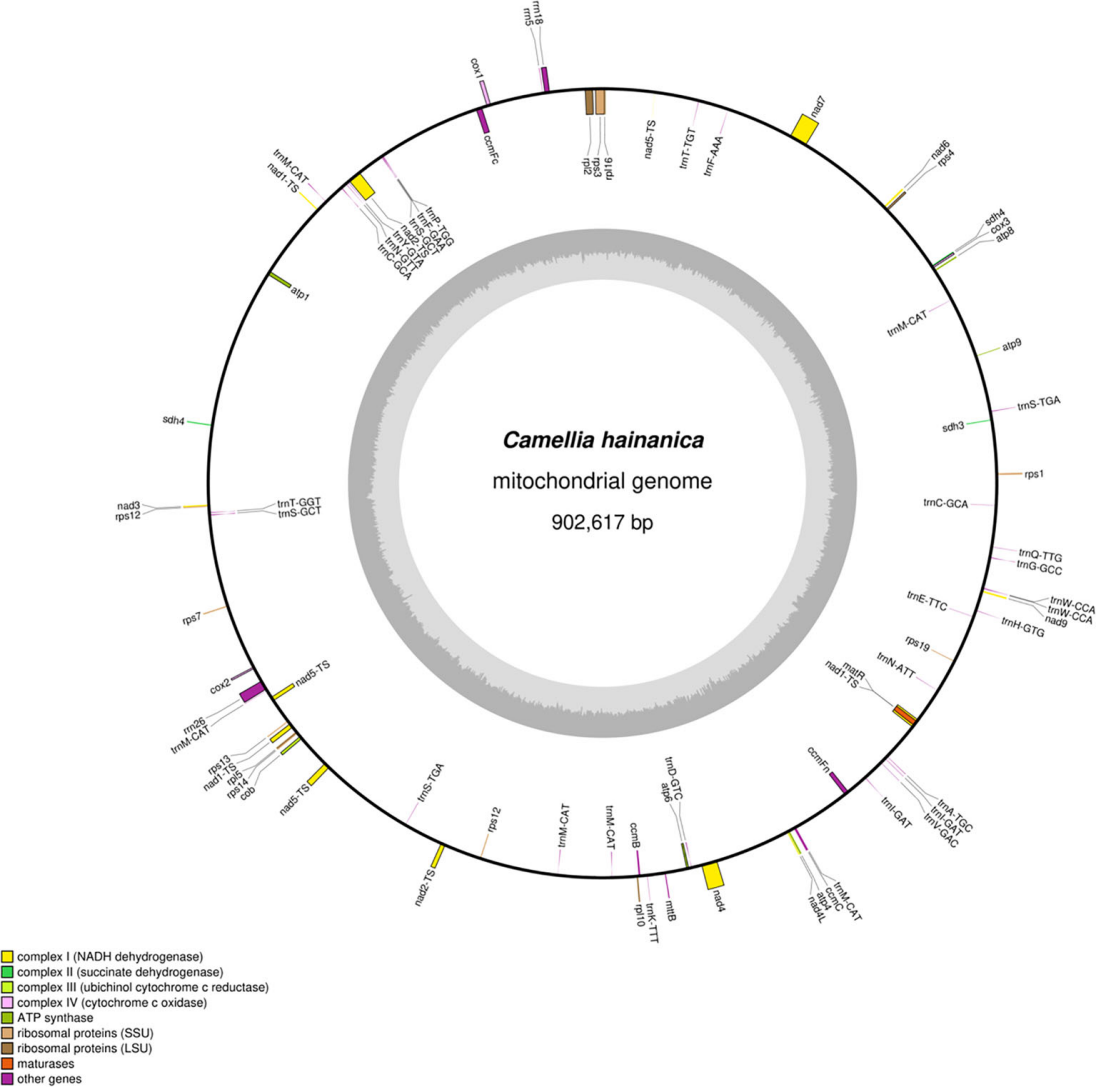
The circular map of *Camellia hainanica* mt genome. The gene map shows 76 annotated genes. These genes are divided into different functional groups, which are distinguished by color - coding on the outer circle. Genes transcribed in the clockwise direction are located on the outer side of the outer circle, while those transcribed in the counter - clockwise direction are on the inner side of the outer circle. The inner circle represents the GC (guanine and cytosine) content in a gray - shaded graphical form.

In this study, *C. hainanica* was sequenced using the Nanopore sequencing platform, obtaining 9,955,548,123 raw data with a mean sequencing read length of 19,911 bp. The N50 read length was 20,944 bp, and the entire mitochondrial genome of *C. hainanica* had a length of 902,617 bp, with a typical circular structure (**Figure 1**). The mitochondrial genome of *C. hainanica* comprised 27.18% of A, 27.02% of T, 22.93% of G, 22.86% of C, and 45.79% of GC. The sample was found to contain 76 genes. Specifically, it includes 40 mRNA molecules, with a combined nucleotide sequence length of 32,826 base pairs (bp) and a guanine-cytosine (GC) content of 43.31%. Additionally, there are 32 tRNA molecules, totaling 2,362 bp in nucleotide sequence length and exhibiting a GC content of 49.87%. The sample also comprises three rRNA molecules, with a combined nucleotide sequence length of 5,650 bp and a GC content of 51.66%.as well as one pseudogene.

In the *C. hainanica* mitochondrial genome (**Table 1**), the *ccmfc*, *rpl2*, *rps3*, *trnA-TGC*, *trnF-AAA*, *trnI-GAT*, *trnS-TGA*, *trnT-GGT*, and *trnT-TGT* genes contain one intron, *nad4* contains two introns,the average length of introns is 744 base pairs (bp). and *nad*1, *nad2*, *nad5*, and *nad7* contain 4 introns,the average length of introns is 1410 base pairs (bp). The genes *rps12*, *sdh4*, *trnC-GCA, trnI-GAT*, *trnS-GCT*, and *trnW-CCA* have two gene copies in the *C. hainanica* mitochondrial gene. The gene *trnM-CAT* has *six* copies in the *C. hainanica* mitochondrial genome.

**Table 1:** Gene profile and organization of the *Camellia hainanica* mt genome.

Group of genes	Gene name
ATP synthase	atp1 atp4 atp6 atp8 atp9
Cytohrome c biogenesis	ccmB ccmC ccmFc* ccmFn
Ubichinol cytochrome c reductase	cob
Cytochrome c oxidase	cox1 cox2 cox3
Maturases	matR
Transport membrance protein	mttB
NADH dehydrogenase	nad1**** nad2**** nad3 nad4** nad4L nad5**** nad6 nad7**** nad9
Ribosomal proteins (LSU)	rpl10 rpl16 rpl2* rpl5
Ribosomal proteins (SSU)	rps1 rps12 rps12 rps13 rps14 #rps19 rps3* rps4 rps7
Succinate dehydrogenase	sdh3 sdh4 sdh4
Ribosomal RNAs	rrn18 rrn26 rrn5
Transfer RNAs	trnA-TGC* trnC-GCA trnC-GCA trnD-GTC trnE-TTC trnF-AAA* trnF-GAA trnG-GCC trnH-GTG trnI-GAT* trnI-GATtrnK-TTT trnM-CAT trnM-CAT trnM-CAT trnM-CAT trnM-CAT trnM-CAT trnN-ATT trnN-GTT trnP-TGG trnQ-TTGtrnS-GCT trnS-GCT trnS-TGA* trnS-TGA trnT-GGT* trnT-TGT* trnV-GAC trnW-CCA trnW-CCA trnY-GTA

*Number of introns

Plant mitochondrial genes differ significantly in size, gene order, and content, so we selected 17 Theaceae mitochondrial genomes for comparative genomic characterization. Six comparative groups were confirmed, namely *Brassica rapa subsp.*, *B. rapa*, *A. thaliana* (Cruciferae), *Aquilaria sinensi* (Thymelaeaceae), *Dalbergia odorifea* (Leguminosae), *Hevea brasiliensi* (Euphorbiaceae), *Olea europaea subsp.* (Oleaceae), and *Cocos nucifea* (Arecaceae), which were then studied to obtain the variability of the mitochondrial genomes of decaploid *C. hainanica* (**Table 2**). The size of the selected mitochondrial genomes ranged from 177,329 to 1,098,121bp.

**Table 2:** Characterization of the mitochondrial genomes of four species of sect.

	*C.hainanica*	*C. huana*	*C. lanceoleosa*	*C.oleifera*	*C. drupifera*
Gene size(bp)	902,617bp	733,752 bp	934,155bp	1,039,838 bp	970,986 bp
Genbank	PV110147	PP975887	PP571817 and PP571818	PP579569	PQ041261 and PQ041262
GC(%)	45.79%	45.73%	45.70%	45.71%	45.73%
Number of gene	76	58	63	63	75
Number of PCGs	40	36	38	38	39
Number of tRNAs	32	20	22	22	32
Number of rRNAs	3	2	3	3	3
Number of pesudo	1				

### 3.2 PCG codon usage bias analysis

**Figure 2:**
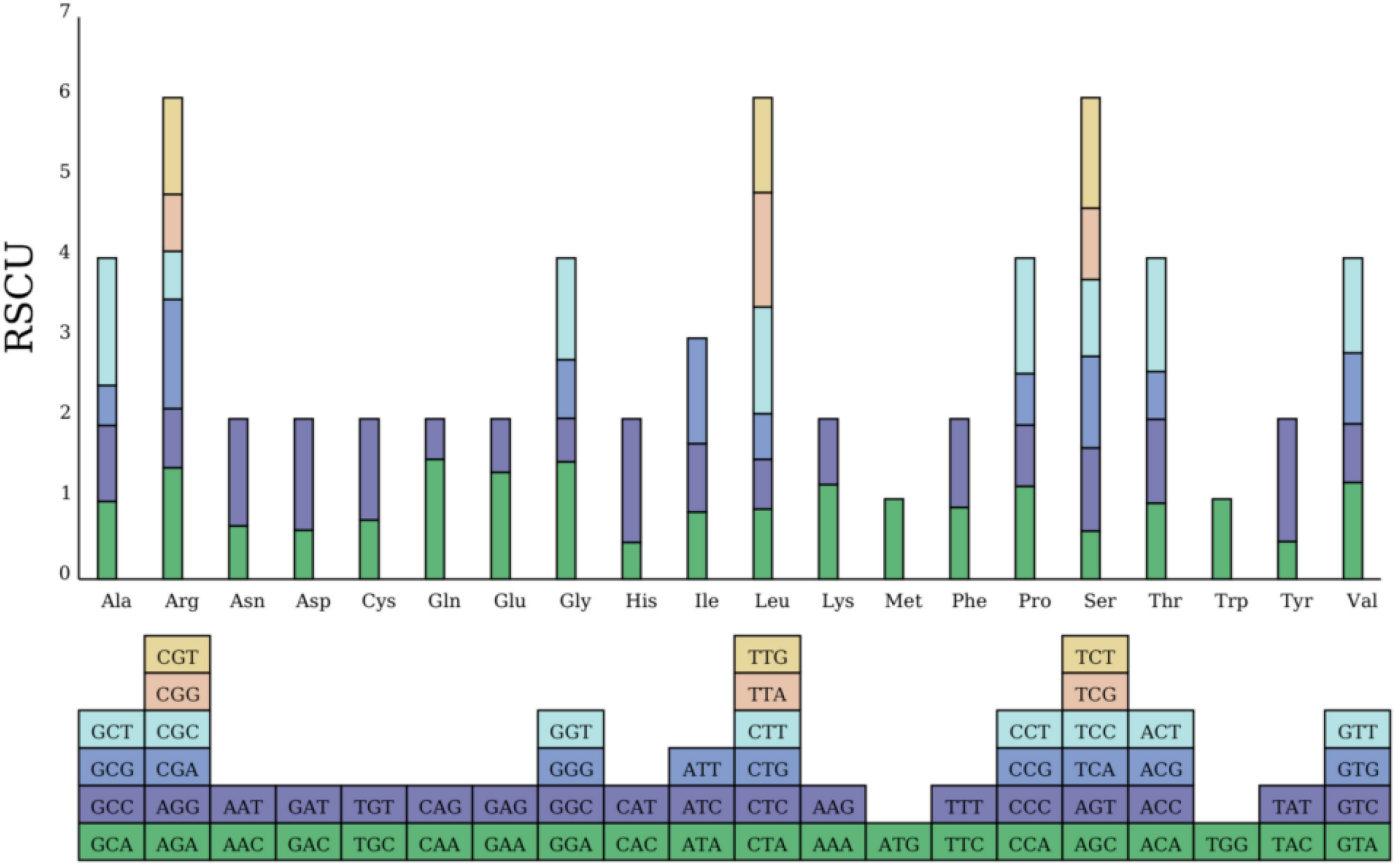
Histogram of relative synonymous codon usage (RSCU). The lower squares represent all codons encoding each amino acid, while the height of the upper bar represents the sum of the RSCU values for all codons.

Codon usage bias can reflect the evolutionary history and environmental adaptations of a species. Three stop codons, UAA, UAG, and UGA, were detected, and C-to-U RNA editing was found in the *ccmFc* gene. The relative synonymous codon usage (RSCU) values of 64 PCGs were also calculated in the *C. hainanica* mitochondrial genome (**Figure 2**). The 64 PCGs encoded 10,687 codons, including the stop codons. Leu (leucine) was the most common amino acid with 1,097 codons, accounting for 10.2%, followed by Ser (serine) with 996 codons, accounting for 9.3%. The rarest amino acid was Ter (stop codon), with 38 codons, accounting for 0.35%. We found 29 codons with RSCU values >1, of which 27 codons (93%) ended in A or U, 1 codon (3.44%) ended in G, and 1 codon (3.44%) ended in C, suggesting that the A/U bias at the third codon is present in *C. hainanica*.

### 3.3 RNA site editing

**Figure 3:**
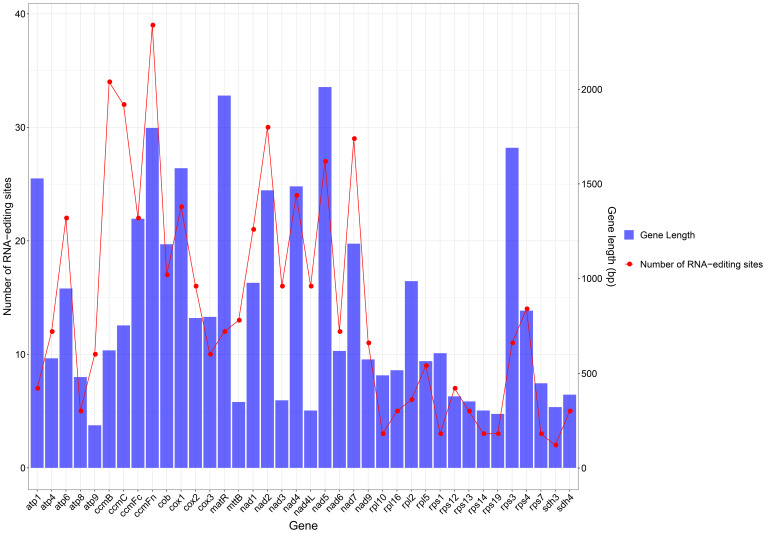
Statistics on the number of RNA editing sites per gene.

In plants, RNA editing is required for gene expression, and C-to-U RNA editing is enriched in mitochondrial and chloroplast genomic species. In this study, 539 RNA editing sites within 64 PCGs were predicted (**Table 3**). From analyzing the relationship between gene length and the number of RNA editing sites, it was found that longer coding sequences had more RNA editing sites. However, there is no absolute linear relationship between these factors (**Figure 3**). From **Table 1**, it can be seen that amino acid changes occurred at all sites, with the main change patterns being as follows: A (Ala)~V (Val), H (His)~Y (Tyr), L (Leu)~F (Phe), P (Pro)~F, P~L, P~S (Ser), Q (Gln)~ *(stop codon), R (Arg)~*, R~C (Cys), R~W (Trp), S~F, S~L, T (Thr)~I (Ile), and T~M (Met). Among these patterns, P~L and P~S had the highest frequency of change, followed by T~M and R~W, while Q~* and R~* had the lowest frequency. The hydrophobicity of 30.43% (164 sites) of amino acids remained unchanged after RNA editing, the hydrophilicity of 12.99% (70 sites) of amino acids remained unchanged after RNA editing, 7.61% (41 sites) of amino acids changed from hydrophobic to hydrophilic, and 48.24% (260 sites) of amino acids changed from hydrophilic to hydrophobic, which therefore led to an increase in the hydrophobicity of the protein. 0.74% (4 sites) of the amino acids changed from hydrophilic to stop codons. Many of the amino acid changes triggered by RNA editing introduce more hydrophobic amino acids into the protein structure, thereby altering the hydrophilicity of the protein and playing a key role in maintaining the regulation of mitochondrial gene expression.

**Table 3:** Statistics regarding the changes in the hydrophilic nature of amino acids induced by RNA editing.

Type	RNA-editing	Number	Percentage
hydrophilic-hydrophilic	CAC (H) => TAC (Y)	9	12.99%
	CAT (H) => TAT (Y)	19	
	CGC (R) => TGC (C)	12	
	CGT (R) => TGT (C)	30	
	total	70	
hydrophilic-hydrophobic	ACA (T) => ATA (I)	4	48.24%
	ACC (T) => ATC (I)	2	
	ACG (T) => ATG (M)	7	
	ACT (T) => ATT (I)	4	
	CGG (R) => TGG (W)	32	
	TCA (S) => TTA (L)	77	
	TCC (S) => TTC (F)	34	
	TCG (S) => TTG (L)	52	
	TCT (S) => TTT (F)	48	
	total	260	
hydrophilic-stop	CAG (Q) => TAG (X)	1	0.74%
	CGA (R) => TGA (X)	3	
	total	4	
hydrophobic-hydrophilic	CCA (P) => TCA (S)	8	
	CCC (P) => TCC (S)	10	
	CCG (P) => TCG (S)	3	
	CCT (P) => TCT (S)	20	
	total	41	7.61%
hydrophobic-hydrophobic	CCA (P) => CTA (L)	49	30.43%
	CCC (P) => CTC (L)	8	
	CCC (P) => TTC (F)	8	
	CCG (P) => CTG (L)	36	
	CCT (P) => CTT (L)	22	
	CCT (P) => TTT (F)	14	
	CTC (L) => TTC (F)	5	
	CTT (L) => TTT (F)	12	
	GCC (A) => GTC (V)	1	
	GCG (A) => GTG (V)	7	
	GCT (A) => GTT (V)	2	
	total	164	
	All	539	100%

Type, type of hydrophilicity change; RNA editing, type of RNA editing; Number, number of RNA edits; Percentage, proportion of the sample.

RNA editing can change the PCG start and stop codons. As shown in **Table 1**, the *cox1* and *nad4L* genes use ACG as the start codon, and so it was hypothesized that this may have been changed by RNA editing. The number of RNA editing sites varied considerably from gene to gene, with the largest number of predictions detected in the cytochrome c biogenesis (*ccmB*, *ccmC*, *ccmFn*, and *ccmFc*), and NADH dehydrogenase (*nad5*) genes.

### 3.4 SSR analysis

**Figure 4:**
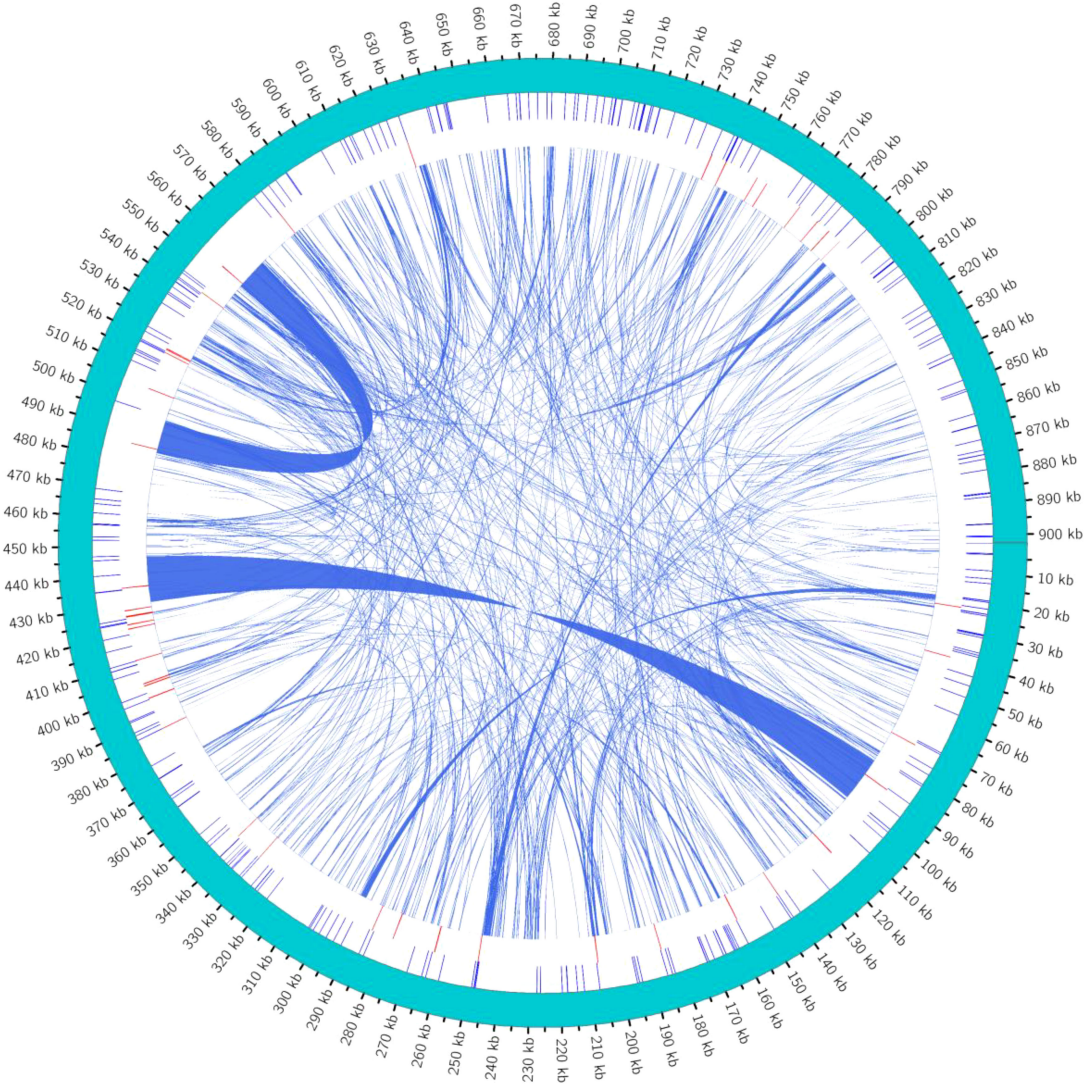
Distribution of repeat sequences across the genome. The outermost circle represents the mitochondrial genome sequence. Moving inwards, they are the simple repeat sequences (in blue), tandem repeat sequences (in red), and interspersed repeat sequences in turn. The simple repeat sequences are a type of tandem repeat sequences that are dozens of nucleotides in length and are composed of repeat units consisting of several nucleotides (1 to 6 nucleotides).

SSRs are characterized by high repeatability, codominant inheritance, uniparental inheritance, and relative conservatism, making them highly efficient molecular markers best suited for species identification and evaluating genetic variation at the population and individual levels.SSRs are stretches of DNA consisting of short unit sequence repeats 1–6 bp in length. Using the MISA tool (Beier et al., 2017), it was found that the minimum number of nucleotide repeats for monomers, dimers, trimers, tetramers, pentamers, and hexamers were 8, 4, 4, 4, 4, and 3 (Zhang et al., 2019), respectively. In this study, a total of *254* SSRs were detected in the mitochondrial genome of *C. hainanica*. The three most common repeat sequences were A/T (93%), AG/TA (44.2%), and AAAG/TTCT (17.3%), with the distribution of these repeat sequences on the genome map shown in **Figure 4**. Among them, tetranucleotide repeat sequences were the most abundant, with 98 in total, accounting for 38.6% of all SSRs. This was followed by dinucleotides (27.6%), of which there were a total of 70. In addition, there were 29 (11.4%) mononucleotides, 37 (14.6%) trinucleotides (Tri-), 16 (6.3%) pentanucleotides, and 4 (1.6%) hexanucleotides (**Table 4**). Among the 254 SSRs, dimers and tetramers were the dominant types of SSR motifs, accounting for 66.2% of all detected SSRs. Tandem repeat sequences are the core repeat units of approximately 1–200 bases. As shown in **Table 5**, 44 tandem repeat sequences with matching of >82% and lengths ranging from 5 to 73 bp were obtained. In addition, there are a total of 691 dispersed repeats exceeding 30 bp, with a total length of 81,690 bp, accounting for 9.05% of the whole mitochondrial genome. The maximum number of repeats ranges from 30 to 65 bp (415 repeats, 60.05%), with three repeats exceeding 1 kb, namely, 16,475, 11,618, and 1,861 bp. The SSR length and number of repeats determine the length and complexity of the repeat base sequences. The above results demonstrate that the *C. hainanica* mitochondrial genome SSR sequences are rich in polymorphisms and can be used for molecular marker development.

**Table 4:** Frequency of identified simple sequence repeat (SSR) motifs.

SSR-Motif		Reputer/number	ssr number	ssr%
Monomer	A	10/7,11/4,12/2,13/1,16/1(15)	29	11.40%
	C	10/1(1)		
	G	10/1(1)		
	T	10/5,11/4,12/2,15/1(12)		
Dimer	AG	5/13,6/3,7/2(18)	70	27.60%
	AT	5/4,6/3,7/3,8/1,9/1(12)		
	CA	5/1(1)		
	CT	5/9,6/3(12)		
	GA	5/6, 6/2(8)		
	GT	5/1(1)		
	TA	5/9, 6/3, 7/1(13)		
	TC	5/5(5)		
Trimer	AAG	4/1, 5/1(2)	37	14.60%
	AGA	4/2(2)		
	AGC	4/3(3)		
	AGT	5/1(1)		
	CAA	4/1(1)		
	CAT	5/1(1)		
	CTT	4/4(4)		
	GGA	4/1(1)		
	GTA	5/1(1)		
	TAA	4/2(2)		
	TAG	4/2(2)		
	TAT	4/2(2)		
	TCC	4/2(2)		
	TCT	4/5(5)		
	TTA	4/3(3)		
	TTC	4/3(3)		
	TTG	4/2(2)		
Tetramer	AAAT, AAGC, AATA, AATG, AGGA, ATAA, ATTG, CAAA, CAAG, CATT, CCAG, CCGA, CGGG, CTAG, CTAT, CTCC, CTTG, GAAT, GACC, GCTT, GGAG, GGCC, GGCC, GTCC, GTGA, TAGA, TAGC, TATT, TCCC, TCGC, TCTT, TGCT, TGGT, TTAT, TTCA, TTGA	3/1(36)	98	38.60%
	AACA, AAGA, AAGG, ACTA, AGCT, ATAG, ATTC, CAAT, CCTT, CTTC, GAAA, GGAA, TCAT, TGAA, TTCC, TTTG	3/2(32)		
	TTTA	3/3(3)		
	TTCT	3/6(6)		
	AGAA	3/7(7)		
	AAAG	3/11(11)		
	TATC, TCTT	4/1(2)		
	TTCT	5/1(1)		
Pentamer	AATCT,ACTAG,ATAAG,ATAGA,ATATA,CCTAT,CTCTA,CTTTC,GAAAT,GGCTT	3/1(10)	16	6.30%
	CTTTA	3/2(2)		
	TTTAT	4/1(1)		
	ATTAA,ATTAG	6/1(2)		
	TATAA	12/1(1)		
Hexamer	AATAGA,CTATCC,GGATAG,TTGCGC	3/1(4)	4	1.60%

**Table 5:** The tandem repeats analysis of *Camellia hainanica* mitochondrial genome.

NO.	Size	Copy	Repeat sequence	Percent matches	Start	End
1	24	3	CCGGCGCAGGCTCAGCAGGAGGGG	97	21887	21958
2	22	2	AGGTTCGGCTAGCTTAGCTATT	86	39259	39302
3	29	1.9	CTTGCATGGACTGAAAGGCTTCCCCTTTA	88	71394	71449
4	26	2	CTTAGGACATACCCAGGCTAATATGA	92	89408	89459
5	61	2	GGTTTTTCAACGTACGATAGCACGGGTTAGCTTGCTTATTTAGAACTAGTGTTCTTAGTTCT	93	117884	118009
6	16	1.9	AGGGTTGTAGAAGTAC	93	140972	141002
7	29	2.2	AACTACCTAGCTACAGGAGGAGAACTACAA	83	157244	157307
8	13	2.3	CTTTCCTTTCTATAG	89	184967	184998
9	24	3	CCGGCGCAGGCTCAGCAGGAGGGG	97	206826	206897
10	18	2.1	TCATATTGATTCTATTTT	90	247687	247723
11	18	2.3	TTGAACTGATTCGAATCC	82	262819	262859
12	20	2	GAAGGGAAGATACCATCCTA	90	277599	277638
13	15	2.1	TCATAGCCGCGAGAGC	88	285307	285339
14	14	2.7	TTCTATTATACTTC	82	331031	331069
15	39	2	AATATCATGATCGGGTCGACCAGGCCAGATCATGAGTGA	97	341180	341258
16	5	12	TATAA	100	386055	386114
17	17	2.1	GAACCCGGCCAGTAAGC	88	397284	397317
18	27	2.4	CCCTTTGAGTAATCTTTAGAATAAAAT	97	401709	401772
19	73	2	GCTTGAACCGTGTCATCGTACGTTGAAAACCCGTGCCATCGTACGTTGGTTCAAGTCTGGTAATGGCGGAAGA	97	402502	402649
20	18	2.1	TTCGATTGGCCTTTCACC	90	410843	410880
21	12	2.1	ATAGGTTCGAAG	100	421825	421849
22	22	2	CGAAGCCTAGAACCAGTGATGA	95	423272	423315
23	12	5.5	TTCTTCGTCCCT	79	425930	425992
24	21	3.7	CTTCTGCTTCTTCGGCCCTTT	80	425923	426003
25	33	2.7	TCATATTCTTTCAATATCTTGTCAATCCTCTCC	84	426116	426205
26	21	2	CGTTTTCTTTTAGAATTGTCT	95	428130	428172
27	26	2	CTTAGGACATACCCAGGCTAATATGA	92	435838	435889
28	16	2.4	GATTCCCTTCCGCTAT	91	485472	485509
29	17	2.6	GGAGTACGAGCTTCGAA	96	504996	505039
30	5	6	ATTAA	100	518373	518402
31	26	2	CAATAGAGAAAGAGGTGTCTGGTGAT	88	519478	519529
32	17	2	AGCGGATCAAAATCGTTG	94	519793	519827
33	15	2.4	AAGTTGCAGAGAGC	90	542376	542408
34	16	2.4	GATTCCCTTCCGCTAT	91	553627	553664
35	6	7.7	TTGCGC	85	580075	580120
36	17	2.2	CTTCTCCTTACTTGGCAG	85	630186	630223
37	20	2	AACCCATTATAATACTAAAG	84	736003	736041
38	5	6	ATTAG	100	741402	741431
39	16	1.9	GTTTGATAGTCTATTCTG	88	753255	753287
40	15	2.5	TAAGAAGAGTAACAG	100	757039	757075
41	19	3.2	ATTCGTTCCTAGAAGAATG	97	770740	770799
42	14	2.1	TCAGCCCTACAAAG	93	778607	778635
43	33	1.9	GCAACTCCAAATCATGGGGACGAATCCCCCCGA	90	783617	783679
44	18	2.1	TCTTTTCTATTAGATTAG	89	788660	788696

### 3.5 Ka/Ks and Pi analyses

In genetics, the use of Ka/Ks ratios to assess the selective pressure of PCGs during the evolutionary dynamics of similar species is essential for reconstructing phylogenetic relationships and studying the evolution of protein-coding sequences between closely related species. Positive selection (Ka/Ks > 1), neutral selection (Ka/Ks = 1), and negative selection (Ka/Ks < 1) are all possible outcomes. This study analyzed the ratios of Ka and Ks in 40 PCGs present in the mitochondrial genomes of *C. hainanica*, *C. hainanica*, *C. chekiangoleosa (PP190481.1)*, *C. lanceoleosa* (PP571818.1), and *C. oleifera* (PP579569.1). The Ka/Ks values of the four common PCGs in *C. hainanica* and *C. hainanica* (NC_086749.1) were zero. The Ka/Ks values of the four common PCGs in *C. hainanica* and *C. chekiangoleosa* (PP190481.1) were also zero. In contrast, only two of the 40 genes common to all species had Ka/Ks values <1, namely *atp6* and *sdh3*, the Ka/Ks values of them are 0.326018 and 0.724737 respectively. This suggests that these two genes have undergone negative selection during evolution, reflecting the tendency of natural selection to remove deleterious non-synonymous mutations.

**Figure 5:**
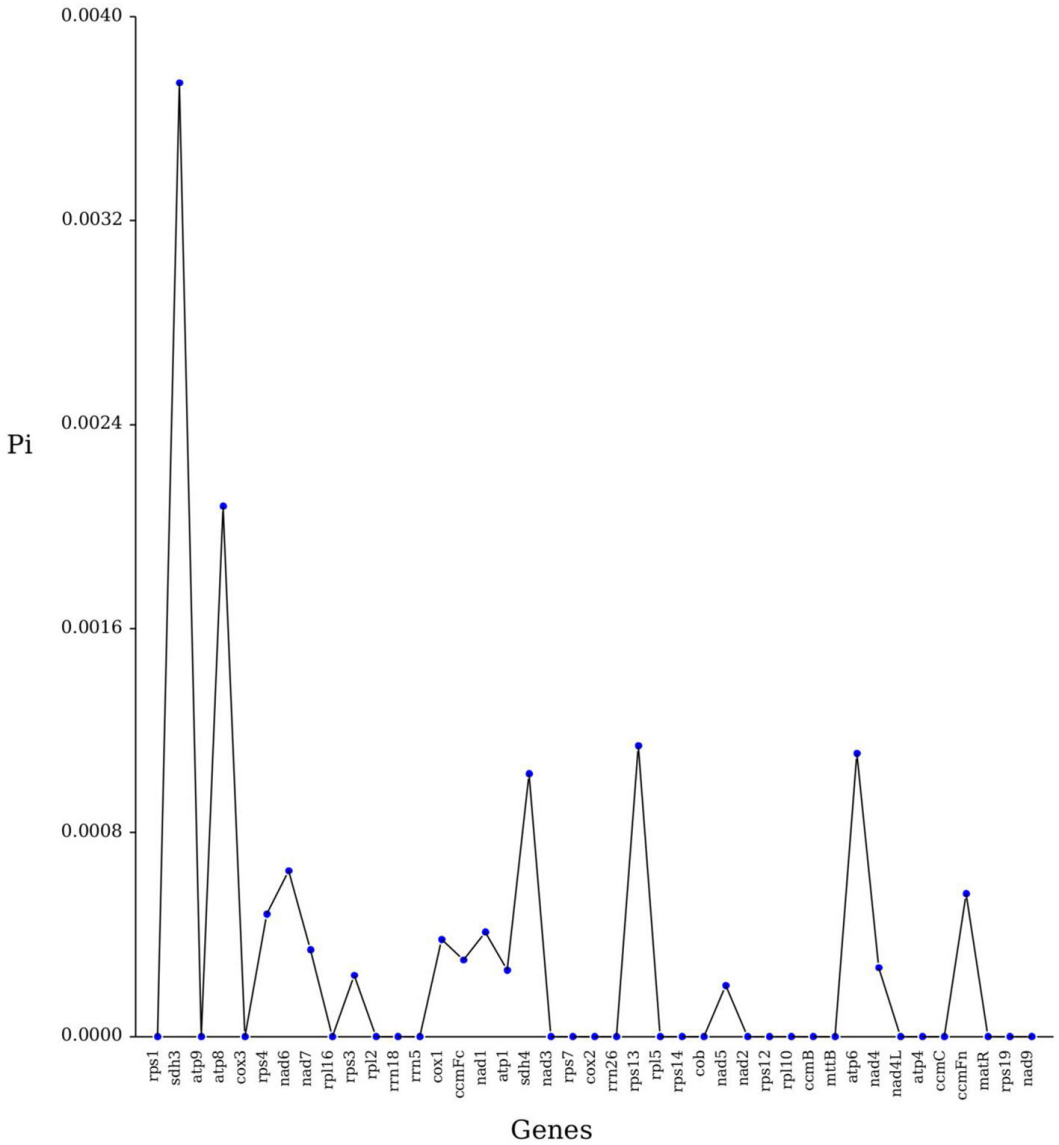
Line graph of gene nucleotide diversity (Pi) values.

Nucleotide diversity (Pi) can reveal the magnitude of variation in the nucleic acid sequences of different species, with regions of higher variation able to provide potential molecular markers for population genetics. This study analyzed the Pi of 40 PCGs of the *C. hainanica* mitochondrial genome. Results showed that the Pi values ranged from 0.0002 to 0.00208, with a mean value of 0.000321 (**Figure 5**). The Pi value of the *sdh3* gene was the largest of these regions at 0.00374. This suggests that the *sdh3* gene can be used as a molecular marker for the mitochondrial genome analysis of *C. hainanica*, followed by the *atp8* gene with a Pi value of 0.00208.

### 3.6 Phylogenetic and collinearity analyses

**Figure 6:**
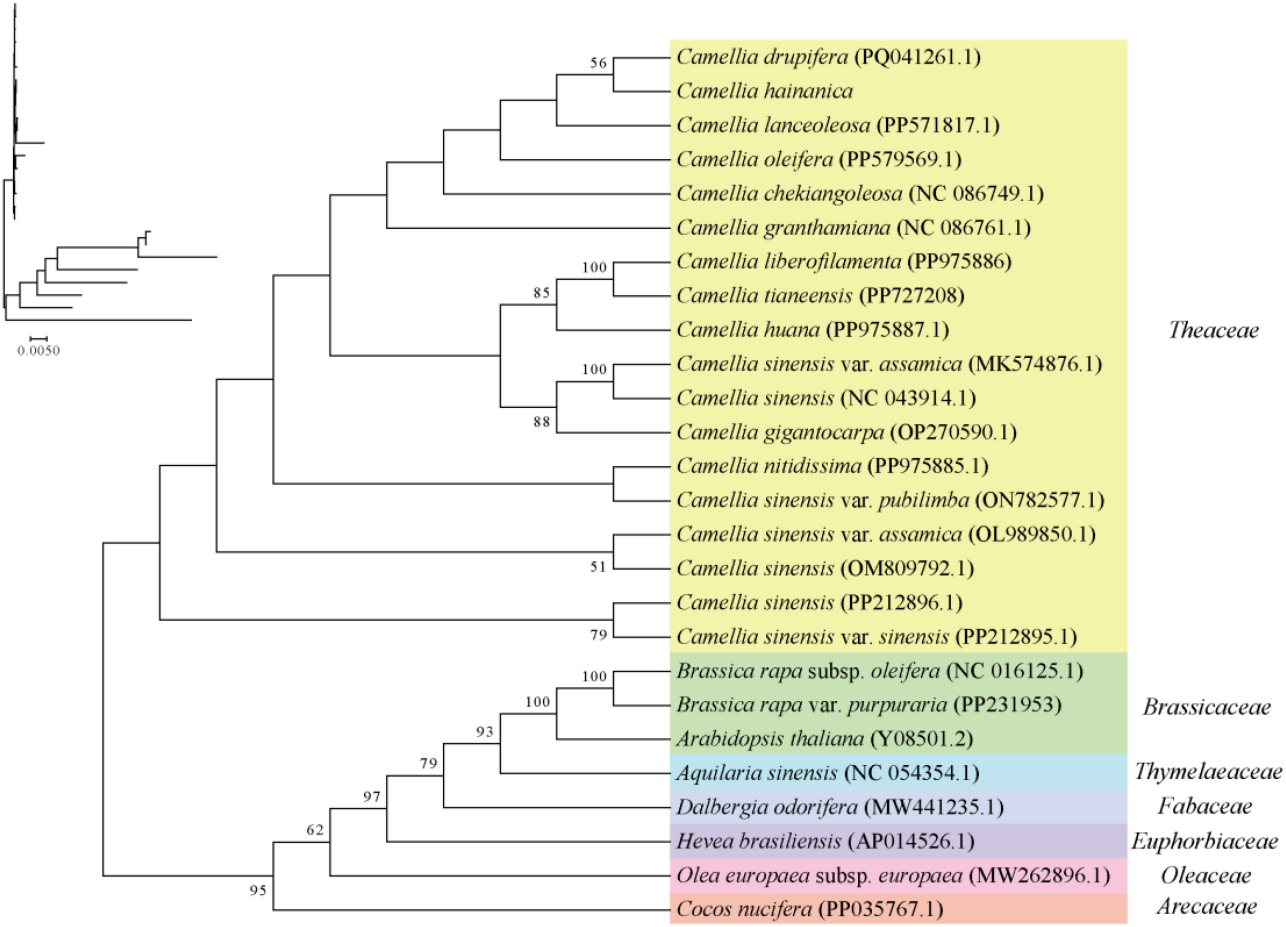
Analysis of mitochondrial phylogeny. The topological structure diagram with branch length information is located in the upper left corner.

This study determined the evolutionary status of the *C. hainanica* mitochondrial genome. A phylogenetic analysis of *C. hainanica* was performed (**Figure 6**), in which the PCGs were determined to be: *atp1*, *atp4*, *atp6*, atp8, atp9, *ccmB*, *ccmC*, *ccmFc*, *ccmFn*, *Cob*, *cox1*, *cox2*, *cox3*, *matR*, *mttB*, *nad1*, *nad2*, *nad3*, nad4, *nad4L*, *nad5*, *nad6*, *nad7*, *nad*9, *rpl10*, *rpl16*, *rpl2*, *rpl5*, *rps1*, *rps12*, *rps13*, *rps14*, *rps19*, *rps3*, *rps4*, *rps7*, *sdh3*, and *sdh4*. The phylogenetic tree was divided into seven groups whose mitochondrial gene sequences were downloaded from GenBank (https://www.ncbi.nlm.nih.gov/genbank/), with the specific genera being *Camellia*(yellow), *Brassica*(green), *Aquilaria*(blue), *Dalbergia*(pale purple), *Hevea*(dark purple), *Olea*(pink), and *Cocos*(orange). Results showed that the species from all families and genera clustered into a single unit, and plants from each different family clustered distinctly with *C. hainanica*. On the phylogenetic tree, the 26 species could be divided into four major branches, with *C. hainanica* and *C. drupifera* clustered closely together, indicating that they had closer phylogenetic affinity.

**Figure 7:**
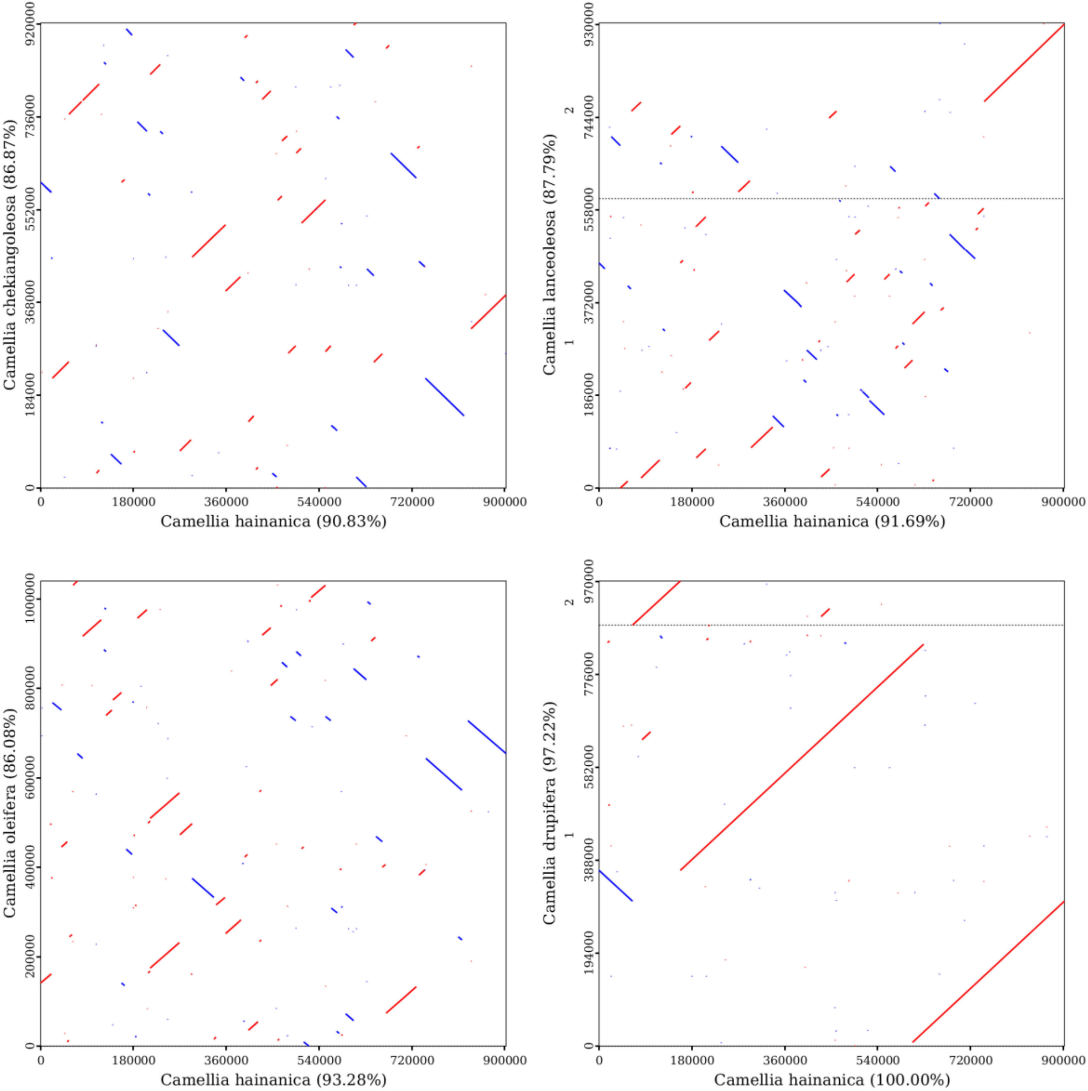
Mitochondrial genome collinearity analysis of the four selected *Camellia* species. The red line indicates forward comparison, the blue line indicates reverse complementary comparison, and the black dotted line represents the dividing line between the two chromosomes.

To better elucidate the conservation of mitochondrial genome evolution between *C. hainanica* and other species in the family Theaceae, a collinearity analysis of the mitochondrial genome sequences was performed (**Figure 7**). Diploid *C. chekiangoleosa*, diploid *C. lanceoleosa*, tetraploid *C. oleifera*, and octaploid *C. drupifera* were selected for collinearity analysis with decaploid *C. hainanica*. Results showed that the decaploid *C. hainanica* and the octaploid *C. drupifera* had a longer diagonal and good collinearity at the mitochondrial structure level, with a collinearity value of 97.22%, suggesting that the genomes were relatively conserved between the two species in terms of the type, order, and direction of genes, implying that the species share a more recent common ancestor.

### 3.7 Analysis of mitochondrial and chloroplast homologous fragments

**Figure 8:**
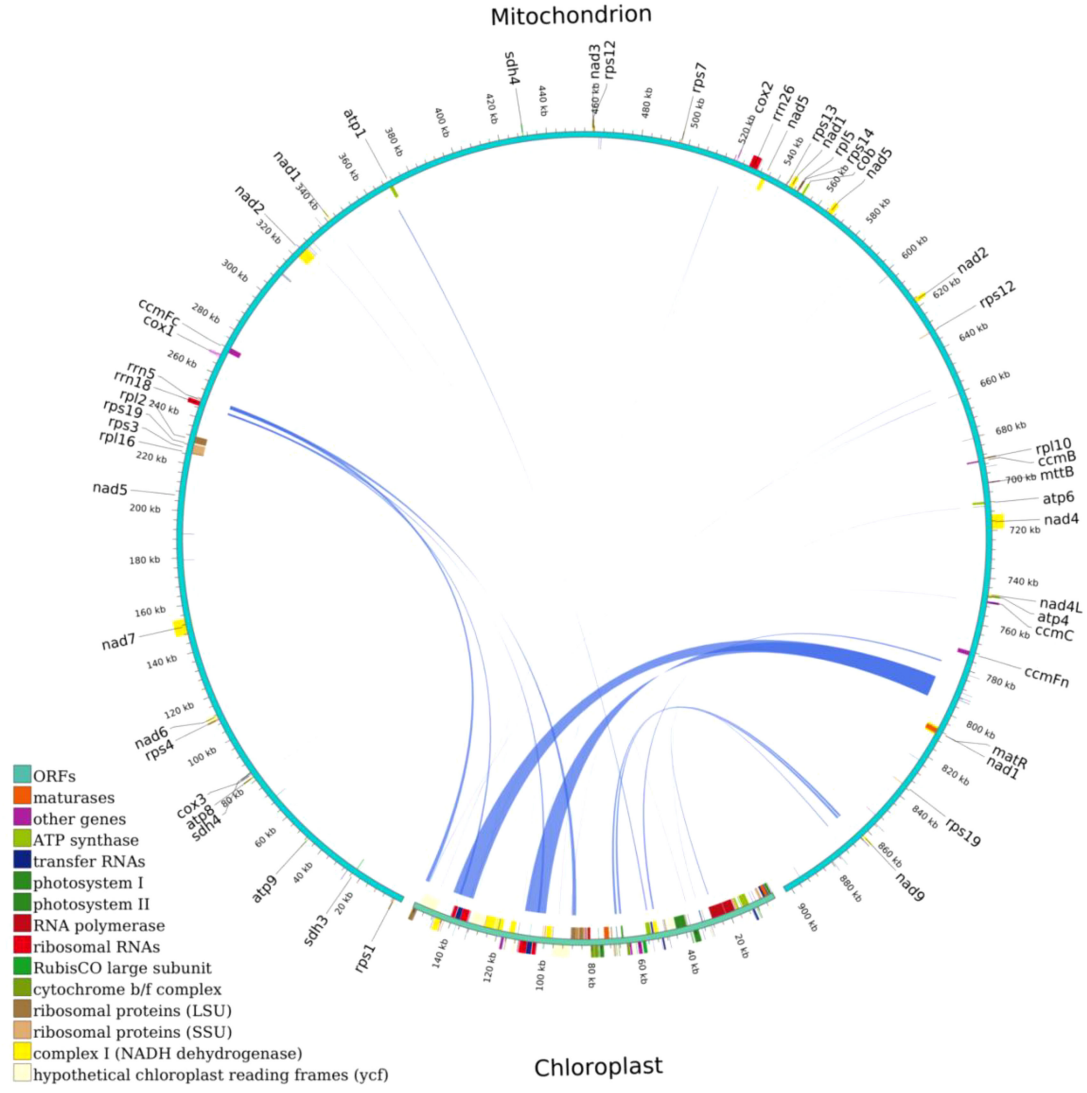
Homologous fragments of chloroplast and mitochondrial sequences. Chloroplasts are chloroplast sequences, while all others are mitochondrial sequences. Genes from the same complex are represented by squares of the same color, with squares in the outer and inner circles indicating genes on the positive and negative strands, respectively, and mid-line connections indicating homologous sequences.

The total length of the chloroplast homologous sequences was 27,468 bp, accounting for 17.5% of the entire chloroplast genome (156,999 bp), while the total size of the mitochondrial homologous sequences was 16,778 bp, accounting for 1.86% of the entire mitochondrial genome (902,617 bp). In total, 34 homologous fragments were found with a total length of 29,841 bp, of which the longest transferred fragment was 9,572 bp, while the shortest fragment was 32 bp (**Figure 8**). The transfer pathway of the fragments may be first from the chloroplast to the nucleus, and then to the mitochondria. Nine genes were highly similar to mitochondrial genes, namely *trnV-GAC, trnI-GAT*, *trnA-TGC*, *trnW-CCA*, *rrn18*, *trnD-GTC*, *trnM-CAT*, *trnN-GTT*, and *ccmC*. These genes may have originated from the mitochondrial genome. Thirty-one genes that were highly similar to chloroplast genes (the 31 genes *rps12*, *rrn4.5*, *rrn23*, *trnA-UGC*, *orf42*, *trnI-GAU*, *rrn16*, *trnV-GAC*, *rpl2*, *rpl23*, *psbJ*, *psbL*, *psbF*, *psbE*, *petL*, *petG*, *trnW-CCA*, *trnP-UGG*, *ndhJ*, ndhK, atpE, atpB, *rpoB*, *psbC*, *trnD-GUC*, *trnI-CAU*, *ycf2*, *trnN-GUU*, *trnM-CAU*, *ndhA*, and *psbB*) were likely to have transformed from the chloroplast genome, whereas only partial sequences of these genes were identified in the mitochondrial genome (**Table 6**). Most of the transferred genes are tRNA genes, which are much more conserved in the mitochondrial genome than in the PCG during evolution.

**Table 6:** Chloroplast genome and mitochondrial genome comparison results.

NO	Identical	Length	Mismatches	Gap	Start of alignment in query	End of alignment in query	Start of alignment in subject	End of alignment in subject	Gene(cp)	Gene(mt)
1	99.342	9572	8	7	133762	143315	796675	787141	rps12;rrn4.5;rrn23; trnA-UGC;orf42;trnI-GAU;rrn16;trnV-GAC	trnV-GAC;trnI-GAT;trnA-TGC
2	99.342	9572	8	7	100337	109890	787141	796675	rps12;trnV-GAC;rrn16; trnI-GAU;trnA-UGC;orf42;rrn23;rrn4.5	trnV-GAC;trnI-GAT;trnA-TGC
3	99.467	1689	1	1	155251	156939	249545	247865	rpl23;rpl2	
4	99.467	1689	1	1	86713	88401	247865	249545	rpl2;rpl23	
5	87.169	982	82	19	66276	67217	866044	865067	psbJ;psbL;psbF;psbE	
6	85.504	1021	85	32	68281	69295	864681	863718	petL;petG;trnW-CCA; trnP-UGG	trnW-CCA;trnW-CCA
7	81.926	758	99	19	51363	52105	779260	779994	ndhJ;ndhK	
8	86.337	505	38	9	54541	55027	368120	368611	atpE;atpB	
9	81.971	416	41	18	25898	26287	871628	871221	rpoB	
10	74.045	890	174	42	139520	140383	245971	245113	rrn16	rrn18
11	74.045	890	174	42	103269	104132	245113	245971	rrn16	rrn18
12	95.918	147	6	0	36637	36783	340240	340386	psbC	
13	91.275	149	13	0	31883	32031	709660	709808	trnD-GUC	trnD-GTC
14	92.913	127	9	0	154860	154986	660602	660476	trnI-CAU	trnM-CAT
15	92.913	127	9	0	88666	88792	660476	660602	trnI-CAU	trnM-CAT
16	88.889	126	8	2	149246	149371	517689	517570	ycf2	
17	88.889	126	8	2	94281	94406	517570	517689	ycf2	
18	90.517	116	4	1	108480	108595	654611	654503	rrn23	
19	90.517	116	4	1	135057	135172	654503	654611	rrn23	
20	96.429	84	2	1	132349	132431	327451	327368	trnN-GUU	trnN-GTT
21	96.429	84	2	1	111221	111303	327368	327451	trnN-GUU	trnN-GTT
22	97.368	76	2	0	88717	88792	750548	750473	trnI-CAU	ccmC;trnM-CAT
23	97.368	76	2	0	154860	154935	750473	750548	trnI-CAU	ccmC;trnM-CAT
24	93.506	77	5	0	54227	54303	337154	337230	trnM-CAU	trnM-CAT
25	100	42	0	0	122881	122922	787772	787813	ndhA	
26	90.566	53	5	0	150673	150725	590443	590391	ycf2	
27	90.566	53	5	0	92927	92979	590391	590443	ycf2	
28	90.385	52	1	3	155041	155092	80066	80019		
29	90.385	52	1	3	88560	88611	80019	80066		
30	97.297	37	0	1	144192	144228	100452	100417		
31	97.297	37	0	1	99424	99460	100417	100452		
32	97.059	34	1	0	76213	76246	84470	84437	psbB	
33	97.059	34	1	0	76213	76246	430900	430867	psbB	
34	96.875	32	1	0	31989	32020	598206	598175	trnD-GUC	

## 4 Discussion

*C. hainanica* is commonly found as a decaploid and octaploid species with large genome data and a complex structure. With advances in sequencing methods, this study obtained more accurate genome assembly sequences of the *C. hainanica* mitochondrial genome (902,617 bp), which has a typical circular structure and is larger than the mitochondrial genomes of most known plants. The GC content was evolutionarily conserved at 45.79%, which is higher than sunflower (45.22%), mango (44.66%), and Purpuraria (*Brassica*) (45.23%), all of which are high levels found in higher plants (Makarenko et al., 2021; Niu et al., 2022; Gong et al., 2024). The functional classifications of protein-coding genes within the mitochondrial genome are relatively conserved across species, and their sequences exhibit a high degree of conservation. This suggests that closely related species maintain a high degree of consistency in the composition of their mitochondrial genes. However, evolutionary events such as gene rearrangements, losses, or duplications can introduce variability. Consequently, even among species with close genetic relationships, differences in the number of genes and their arrangement within the mitochondrial genome may still be observed (Clifton et al., 2004; Ogihara, 2005). The coding regions of the genome are more conserved than the noncoding regions, and the noncoding regions are also the main source of mitochondrial genome variation (Christensen, 2013). The intergenic region of the mitochondrial genome mainly comprises repeat sequences, chloroplast genome homologous sequences, and contains tandem repeat sequences, dispersed repeat sequences, and SSRs. These are all widespread in the mitochondrial genome (Guo et al., 2017), are essential for the intermolecular recombination of the mitochondrial genome, and are often considered to be the main cause of mitochondrial genome variability (Dong et al., 2018). Most PCGs start with a typical ATN codon (Lin et al., 2017), and some genes contain one or more introns that may play an important role in regulating gene expression.

Ka/Ks ratios are important for assessing the impact of environmental stresses on plants during evolution and can reveal the effects of genetic changes on the phenotypes of different seed plants. During plant evolution, most mitochondrial genes with Ka/Ks <1 exhibited negative selection, while a few genes with Ka/Ks >1 exhibited positive selection (Xu et al., 2021). It was concluded from the study of the mitochondrial genome of *C. hainanica* that *sdh3* and *atp6* exhibited negative selection, suggesting that these genes may be selected for use in future studies of gene selection and phylogeny in species from the genus *Camellia.* The size and structure of the mitochondrial genome of plants have changed significantly, while functional genes remain conserved. Pi analysis reflects variation in nucleotide sequences between species. Results showed that the Pi value of *sdh3* was the largest among these regions, indicating that the *sdh3* gene can be used as a molecular marker for *C. hainanica* mitochondrial genome analysis.

PCG is usually encoded from the start codon (ATG) to the stop codon (UAA, UAG, and UGA), with the distribution of the amino acid composition found to be consistent with that of *A. thaliana*. Codon usage bias refers to the presence of synonymous codons in a non-random manner across different species (Li et al., 2023a). The analysis of codon usage patterns helps to elucidate the molecular mechanisms of biological adaptations and to explore evolutionary relationships among species (Ding et al., 2023). Previous studies have shown that there was a bias toward A/U at the ends of codons in plant mitochondrial genomes, with *93%* of codons in the *C. hainanica* mitochondrial genome ending in A or U, which may be the result of natural selection, mutational pressure, and genetic drift (Bulmer, 1991). In addition, leucine was found to be the most commonly used amino acid, which is consistent with *Acer truncatum* Bunge (Ma et al., 2022).

The number of RNA editing sites varies from plant to plant and is commonly found in the mitochondrial genomes of gymnosperms and angiosperms. This study obtained *539* RNA editing sites within 64 PCGs of *C. hainanica*, which is lower than that of *Taxus cuspidata*(974) *Ginkgo biloba*(1306)and *Pinus taeda*(1179) (Kan et al., 2020), and much higher than that of *okra* (85) (Li et al., 2022) and *Melia azedarach L*(356) (Hao et al., 2024) The selection of *RNA* sites in *C. hainanica* showed a high degree of compositional bias. Most RNA editing sites are C-to-U transitions, and most amino acids are converted to hydrophobic amino acids during RNA editing, increasing the hydrophobicity of the edited proteins and thereby increasing the stability of the proteins. Hydrophilic amino acids are distributed on the surface of the protein molecule, whereas hydrophobic amino acids are mainly distributed in the interior of the molecule. The correlation between hydrophilic and hydrophobic amino acids can be used to determine general trends in protein folding. The identification of these RNA editing sites provides important clues for future studies on the evolution of gene function and the prediction of new codons, and can help to provide a better understanding of gene expression in plant mitochondrial genomes.

Repeat sequences are essential for intermolecular gene recombination and have been widely used to confirm phylogenetic relationships, conduct genetic diversity studies, and achieve species identification due to the high variability and recessive inheritance of SSRs (Ping et al., 2021). The mitochondrial genome of *C. hainanica* contains 254 SSRs, 93% of which are monomers A or T, a genome that is similar to that of sugarcane, *Diospyros kaki Thunb. ‘Taishuu’* (Ebenaceae), and *Bougainvillea* sp*ectabilis* and *Bougainvillea glabra* (Nyctaginaceae) (Yang and Duan, 2024; Zhang et al., 2023). In addition, 44 tandem repeats and 691 dispersed repeats were found in this study, values which are much larger compared to *B. oleracea* var. *Italica* (broccoli) (Zhang et al., 2022a).

Transfer of DNA between the chloroplast and mitochondrial genomes is frequently observed in plant mitochondria (Straub et al., 2013). In higher plants, the size of transferred DNA varies from 50 kb (*A. thaliana*) to 1.1 Mb (*Oryza sativa subsp. japonica*; japonica rice) (Smith et al., 2011), depending on the plant species. A total of 29,841 bp of chloroplast DNA was transferred to the *C. hainanica* mitochondrial genome, and around 34 fragments were transferred from the chloroplast genome to the mitochondrial genome, containing nine annotated genes, including seven tRNA genes (*trnV-GAC*, *trnI-GAT*, *trnA-TGC*, *trnW-CCA*, *trnD-GTC*, *trnM-CAT*, and *trnN-GTT*) along with *rrn18* and *ccmC*. The transfer of tRNA genes from the chloroplast to the mitochondrial genome is common in angiosperms (Bi et al., 2016). These results are consistent with previous findings, which revealed that tRNA genes are more conserved than PCGs during evolution and that tRNA genes play an integral role in the mitochondrial genome.

In this study, a phylogenetic tree was constructed based on the mitochondrial genomes of 25 plant species, and the whole mitochondrial genome sequence was applied to *C. hainanica* for the first time. The sequenced mitochondrial genome sequences of *Camellia* genus plants and the published mitochondrial genome sequences of six other families were selected for phylogenetic analysis. Results showed that *C. hainanica* was well clustered with the genus *Camellia*, with the classification of the families clearly visible. Of the 25 species, its closest relative was *C. vietnamensis*.

Plant mitochondrial genomes, which are characterized by structural rearrangements, large numbers of genes being lost or gained, mitochondrial to nuclear gene transfer, and very low rates of nucleic acid mutations, provide unique information for phylogenetic analyses and homologous collinearity analyses among plant mitochondria that can reveal relationships and evolutionary histories among different species. Evolutionary analyses showed that the mitochondrial genome of *C. hainanica* experienced frequent genetic recombination events during the evolutionary process, and these colinear regions not only revealed the conserved patterns of the mitochondrial genome but also reflected the evolutionary relationships and evolutionary history among species, providing a new perspective to reveal the phylogeny and the genetic basis of the *C. hainani*ca species.

## 5 Conclusion

This study sequenced and successfully assembled the complete mitochondrial genome of decaploid *C. hainanica* with a typical circular molecular structure. Various genetic aspects of *C. hainanica* were investigated, including its compositional structure, codon preference, RNA editing sites, and repeat sequences, and an integrated alignment analysis was conducted in terms of Ka/Ks and Pi, which revealed the structure of the mitochondrial genome of decaploid *C. hainanica*. Subsequent phylogenetic and collinearity analyses found that decaploid *C. hainanica* was clustered together with *C. vietnamensis* on the phylogenetic tree, suggesting that the two species are more closely related. Horizontal gene transfer of DNA between the mitochondrial and chloroplast genomes was also found in *C. hainanica*, confirming that tRNA genes are genetically conserved over PCGs during evolution. This study provides more comprehensive genetic information on the genome of *C. hainanica*, which is important for revealing the function of the mitochondrial genome and studying the genetic characteristics, evolutionary origin, conservation and utilization, and taxonomic status of this plant family.

## Data Availability

The data presented in the study are deposited in the National Center for Biotechnology Information (NCBI) (https://www.ncbi.nlm.nih.gov) repository, accession number PV110147.
