# Correction: Mitochondrial genome assembly and comparative analysis of decaploid *Camellia hainanica*

**DOI:** 10.3389/fpls.2025.1658310

**Published:** 2025-07-23

**Authors:** Shihui Zhang, Yuyan Zhang, Sheng Luo, Jie Gao, Haiyan Hu, Jinping Liu, Wenqiang Wu, Jian Wang, Xiaolong Huang, Hanggui Lai, Dongyi Huang

**Affiliations:** ^1^ School of Tropical Agriculture and Forestry, Hainan University, Danzhou, China; ^2^ School of Life and Health Sciences, Hainan University, Haikou, China; ^3^ School of Breeding and Multiplication (Sanya Institute of Breeding and Multiplication), Hainan University, Sanya, China

**Keywords:** Decaploid *Camellia hainanica*, mitochondrial genome, comparative analysis, RNA editing, analysis system development

In the published article, there was a mistake in the Author name. An author name was incorrectly written as “Hangui Lai”. The correct spelling is “Hanggui Lai”.

In the published article, there was a mistake in the author designation. A dagger symbol ^(†)^ was used next to the names of “Hanggui Lai and Dongyi Huang”. These two authors were incorrectly written as “Hanggui Lai^1,3^*^†^ Dongyi Huang^3^*^†^”. These two authors were the corresponding authors and did not share first authorship.

The authors apologize for this error and state that this does not change the scientific conclusions of the article in any way. The original article has been updated.

