# Supplementary figures and images for: Mitochondrial genome assembly and comparative analysis of decaploid Camellia hainanica
Source: Front Plant Sci. 2025 Jun 30;16:1556379. doi: 10.3389/fpls.2025.1556379 (PMC12257034; doi:10.3389/fpls.2025.1556379)

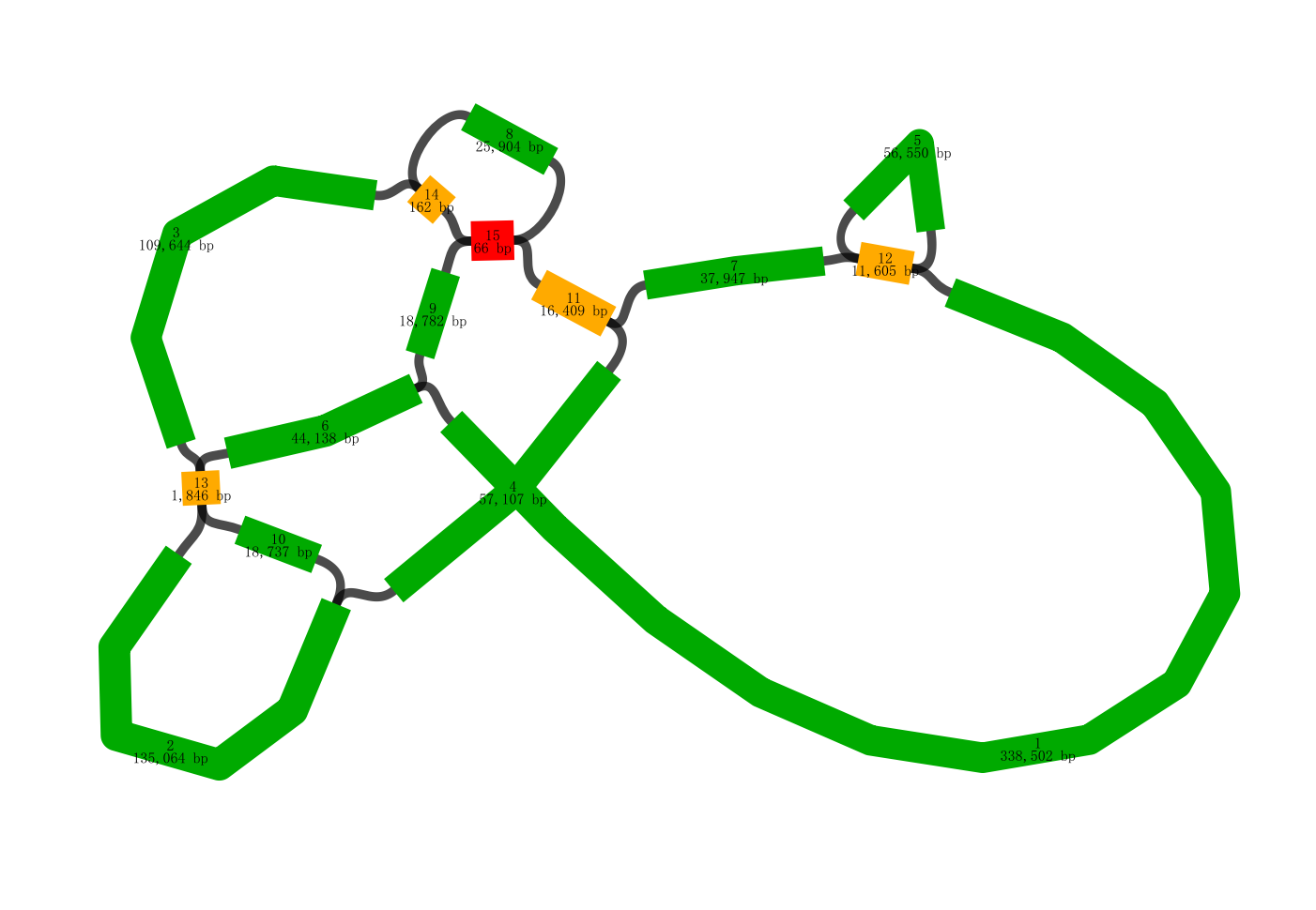

Supplement: Supplementary file 1 [file DataSheet1.zip › Supplementary files/Camellia_hainanica.gfa.png]

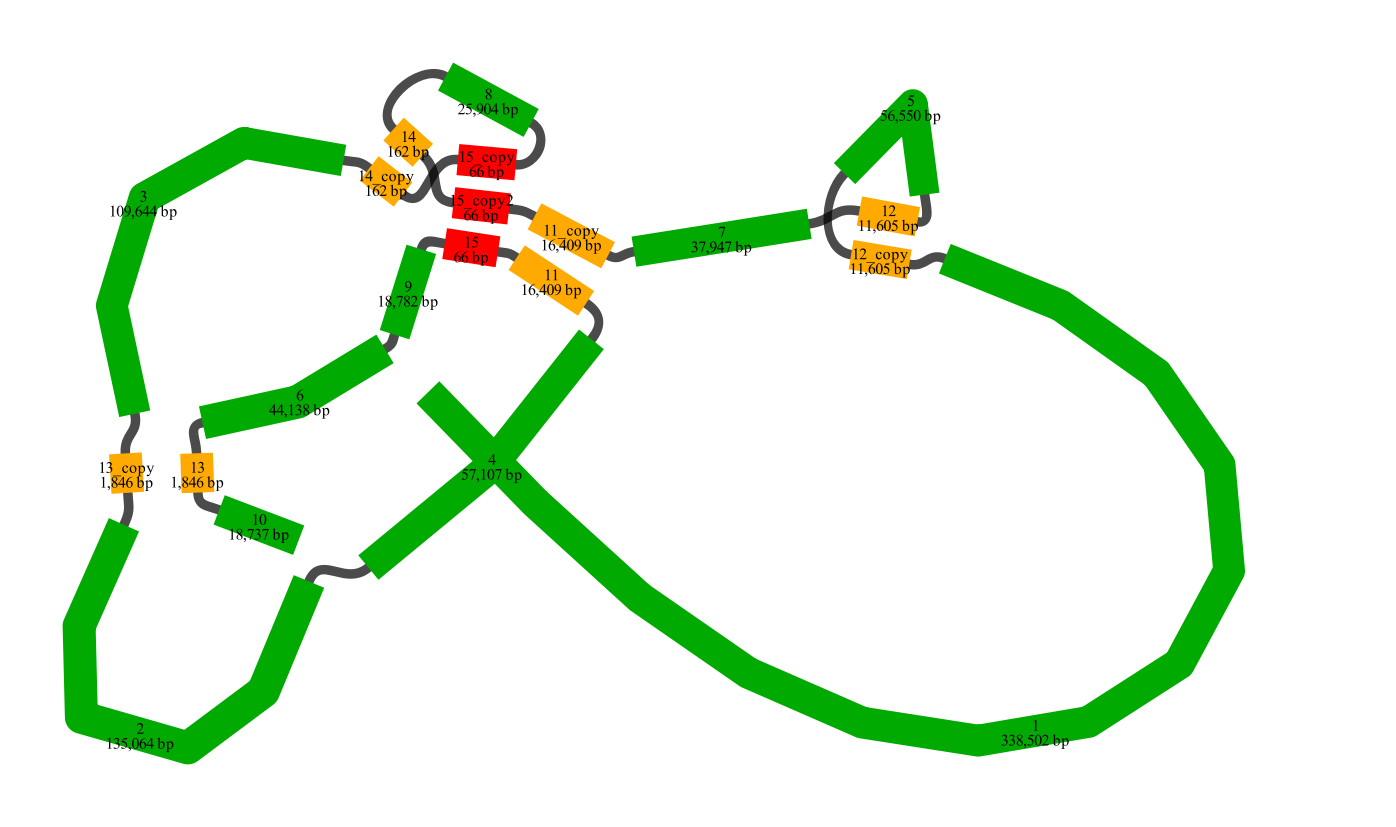

Supplement: Supplementary file 1 [file DataSheet1.zip › Supplementary files/Camellia_hainanica.split.gfa.png]

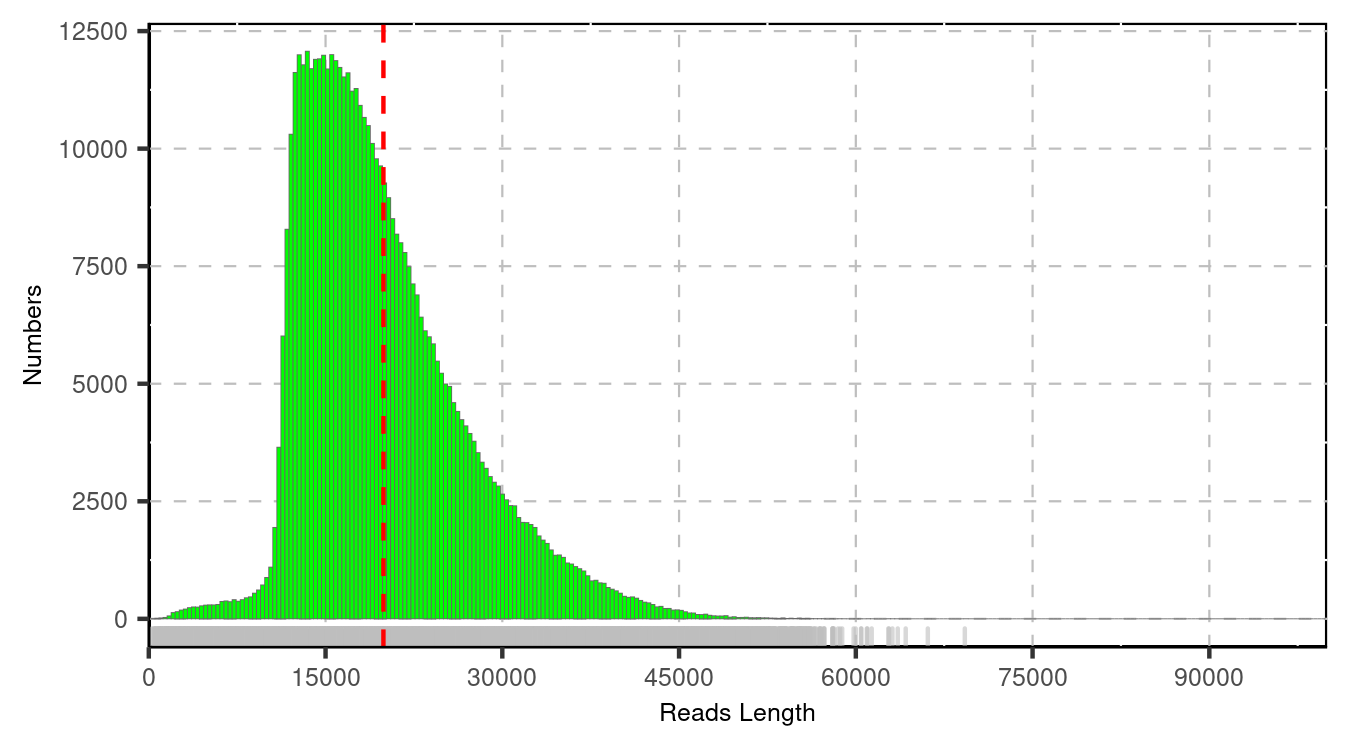

Supplement: Supplementary file 1 [file DataSheet1.zip › Supplementary files/Camellia_hainanica_tgs_reads_length_plot.png]
